# Customized yeast cell factories for biopharmaceuticals: from cell engineering to process scale up

**DOI:** 10.1186/s12934-021-01617-z

**Published:** 2021-06-30

**Authors:** Aravind Madhavan, K. B. Arun, Raveendran Sindhu, Jayaram Krishnamoorthy, R. Reshmy, Ranjna Sirohi, Arivalagan Pugazhendi, Mukesh Kumar Awasthi, George Szakacs, Parameswaran Binod

**Affiliations:** 1 Rajiv Gandhi Centre for Biotechnology, Jagathy, Trivandrum, 695 014 Kerala India; 2 Microbial Processes and Technology Division, CSIR-National Institute for Interdisciplinary Science and Technology (CSIR-NIIST), Trivandrum, 695 019 India; 3 Stelis Biopharma, Bangalore, India; 4 Post Graduate and Research Department of Chemistry, Bishop Moore College, Mavelikara, 690 110 Kerala India; 5 Department of Chemical & Biological Engineering, Korea University, Seoul, 136713 Republic of Korea; 6 School of Renewable Energy, Maejo University, Chiang Mai, 50290 Thailand; 7 College of Medical and Health Science, Asia University, Taichung, Taiwan; 8 College of Natural Resources and Environment, Northwest A & F University, Yangling, Xianyang, 712100 Shaanxi China; 9 Department of Applied Biotechnology and Food Science, Budapest University of Technology and Economics, Szent Gellert ter 4, Budapest, 1111 Hungary

**Keywords:** Therapeutic proteins, Yeast, Secretion signal, Humanized yeast, Glycosylation

## Abstract

The manufacture of recombinant therapeutics is a fastest-developing section of therapeutic pharmaceuticals and presently plays a significant role in disease management. Yeasts are established eukaryotic host for heterologous protein production and offer distinctive benefits in synthesising pharmaceutical recombinants. Yeasts are proficient of vigorous growth on inexpensive media, easy for gene manipulations, and are capable of adding post translational changes of eukaryotes. *Saccharomyces cerevisiae* is model yeast that has been applied as a main host for the manufacture of pharmaceuticals and is the major tool box for genetic studies; nevertheless, numerous other yeasts comprising *Pichia pastoris, Kluyveromyces lactis, Hansenula polymorpha, and Yarrowia lipolytica* have attained huge attention as non-conventional partners intended for the industrial manufacture of heterologous proteins. Here we review the advances in yeast gene manipulation tools and techniques for heterologous pharmaceutical protein synthesis. Application of secretory pathway engineering, glycosylation engineering strategies and fermentation scale-up strategies in customizing yeast cells for the synthesis of therapeutic proteins has been meticulously described.

## Background

Several important natural compounds have been used as pharmaceuticals, flavourings, nutraceuticals, and fragrances. Most of these molecules are either plant-derived, having difficult extraction process and less yield, or synthesised by organisms that cannot be simply employed in industrial level synthesis [1]. In spite of the attempts to improve the production efficiency of these natural synthesisers, there are still restricting features for industrial synthesis such as small rate of growth and unpredictability in yield. Recombinant protein synthesis in microbes has been attaining large consideration because of numerous benefits such as quicker, more cost-effective, easy gene manipulations, very easy downstream processes [2].

The classical baker’s yeast *Saccharomyces cerevisiae* is among the extensively studied eukaryotic yeast and most commonly used heterologous and homologous host for biopharmaceutical synthesis, gene manipulation and protein production [3]. In addition to *S. cerevisiae*, several non-conventional yeast species like *Hansenula polymorpha*, *Pichia pastoris*, *Yarrowia lipolytica*, *Schizosaccharomyces pombe*, and *Kluyveromyces lactis* have been developed as substitute hosts for the synthesis of heterologous proteins [4].

The complete genome of *S. cerevisiae* was published in 1996 [5], and the full sequences of genome of many other alternate yeasts are also presently accessible in the public domain [5] enabling the analysis of various omics generated functional genomics data. Functional genomics and system biology could facilitate a widespread understanding of yeast molecular physiology, which could aid to delineate potential drawbacks in protein manufacture on a comprehensive level. Additionally, in the past few years synthetic biology and CRISPR based genome manipulation techniques emerged as a revolutionary techniques to expedite the rational design process of yeast cell factory development [6].

As a recombinant production host, yeast has been traditionally applied to synthesise a large variety of compounds such as, aromatics, terpenoids, sterols, alcohols, sugar derivatives, citric acid, lactic acid, organic and fatty acids, terpenes, peptides and several medically important therapeutic proteins [7]. Among these, therapeutic biopharmaceuticals are one of the fastest-developing multibillion dollar industries. In the year 2015, the vaccination trade has spawned approximately 28 billion US dollars and is predicted to reach up to 39 billion in 2022. The trade of other therapeutically components accounts for 163 billion US dollars in 2016 [8]. Table 1 enlisted the major therapeutic proteins produced in yeast. It is assumed that this market potential has great impact on dynamic growth. The global share of therapeutic proteins like anti-tumour necrosis factor antibodies, monoclonal antibodies cancer proteins, hormones like insulin and its analogues, growth hormone is 25% compared to other commercial pharmaceutical products and possess 40% of total trades of pharmaceuticals [9].

**Table 1:** Representative list of therapeutic proteins produced in yeast

Host	Therapeutic protein	Yield	Reference
*P. pastoris*	Insulin	3 g/L Insulin precursor	[122]
*S. cerevisiae*	IFNα2b	15 mg/L	[142]
*Y. lipolytica*	IFNα2b	425 mg/L	[143]
*P. pastoris*	Hepatitis B antigen	7 g/L	[144]
*S. cerevisiae*	Glucagon like peptide 2	–	[145]
*K. lactis*	Human interferon β	–	[40]
*P. pastoris*	Human granulocyte–macrophage colony-stimulating factor	285 mg/L	[146]
*P. pastoris*	Human serum albumin	92.29 mg/L	[147]
*H. polymorpha*	HBV surface antigen	250 mg/L	[148]
*H. polymorpha*	Granulocyte colony stimulating factor (GCSF)	–	[26]
*H. polymorpha*	Rotavirus VP6 protein (RV VP6)	3350.71 mg/L	[149]
*H. polymorpha*	HPV type 16 L1-L2 chimeric protein (SAF)	132.10 mg/L	[25]

Conversely, yeast can efficiently resolve issues related to low heterologous pharmaceutical product yield in other expression hosts like *E. coli* and mammalian system. Since it is capable to produce several heterologous pharmaceutical proteins, and offers less susceptibility to contaminations by phage and improved secretion efficiency than bacteria. Furthermore, yeast has very efficient tolerance to low pH, fermentation inhibitors and is generally recognised as safe [10]. More significantly, yeast efficiently modifies its recombinant proteins post transnationally.

The current review describes the status of gene manipulation strategies for recombinant therapeutic protein production in yeast.

## Promoter engineering

Proficient transcription is a key stage in regulating the expression of gene. Consequently, well-studied inducible or constitutive promoters with robust transcription strength are normally applied to attain overexpression of heterologous proteins. The widely studied and efficient constitutive *GPD* and *TEF1* promoters have been applied for robust synthesis of heterologous proteins in *S. cerevisiae* [11]. It is notable that in the scenario of production of secretory heterologous protein, robust protein induction and flux might somewhat lead to lowered efficiency of secretion because of endoplasmic reticulum accumulation of unfolded proteins, as observed in the synthesis of various clinically relevant antibodies, *α*-amylase, and insulin precursor in *S. cerevisiae* [12]. Therefore, a series of promoters having various rate of transcriptional action might be beneficial to attain optimized secreted expression. Conversely, native promoters might not give varying strength of transcription thereby restricting the capability to fine-regulating gene expression.

Presently, computer aided machine learning is largely applied for engineering of promoter in *E. coli*. In *S. cerevisiae*, libraries of promoter were constructed based on widely used wild type promoters, comprising *PTDH3* and *PZEV*. Moreover, researchers have constructed models to predict promoter strength directly from sequence. Deep learning technology including convolutional neural networks (CNNs) has performed well on genomics modelling tasks. The transcription strength of promoters from the library was assessed using CNN model. The transcriptional strength of a synthetic promoter which is assessed from the *TDH3* promoter enhanced the activity by 37% and the transcriptional action of a mutant *ZEV* promoter also enhanced by induction using β-estradiol [13].

Another synthetic promoter was effectively synthesised and combined with *P. pastoris AOX1* cis-regulatory region using computational strategies and selection of libraries, and these promoters core can be applied to several species of yeast, comprising *S. cerevisiae* and *P. pastoris* [14]. Promoter length of *E. coli* is normally below 100 bp, however yeast promoters have size above 100 bp. The larger nucleotide base pair not only decreases the efficacy of metabolic construction of pathway, but also obstructs the pathway regulation. The synthesis of promoters with minimal elements could overcome these inefficiencies. The removal of non-essential promoter regions is one technique for promoter construction with minimal essential regions [15]. *PTEF1* from *S. cerevisiae* was modified by deleting non-essential promoter region and the results showed that 69 bp core elements can perform transcriptional activities. Several promoters were constructed by combining minimal essential elements and upstream activating sequence which resulted a 80% transcriptional *PTEF1* activity [15]. Conversely, the modified promoters developed by this technique possess disadvantage from homologous recombination since these promoters might still have sequences from endogenous promoters. Mutagenesis by saturation might be a desirable technique for synthesising minimal promoters [16]. Promoter engineering was done in *S. cerevisiae*, to attain minimal core promoter elements with size ranging from 20 to 30 bp among TATA box and TSS were shortlisted for saturation mutagenesis. By joining the minimal core promoter elements and UASs, several minimal promoters were constructed and the transcriptional level of many of them attained 70% of the native PTDH3, with only a very small percentage of its original length.

Robust and regulated promoters are an indispensable tool for enhanced level recombinant protein synthesis [17]. There has been an increasing demand in the construction of synthetic promoters which drives enhanced heterologous protein expression, enhance protein folding and tightly regulatable transcription profile [18]. *P. pastoris* synthesised up to 22 g/L intracellular and 15 g/L extracellular proteins with the help of highly regulated, robust methanol inducible *AOX1* promoter [19]. A promoter library by deletion and duplication of putative transcription factor-binding sites within the *AOX1* promoter (P_*AOX1*_) sequence was constructed. This promoter library provided an approximate activity range between 6 and 160% compared to the wild type promoter activity. After extensive characterization of the promoter library by employing a green fluorescent protein variant gave 5–150% of activity compared to native P_*AOX1*_ driven recombinant protein production. [20]. Some point mutations and deletions of promoter region caused in altered transcriptional regulation of these variant promoters and was found to be reasonably dynamic when glucose was exhausted, lacking necessitating the methanol inducer [20]. This de-repression by glucose depletion bettered the use of promoter with full functional elements in definite uses particularly when several replicas of the expression constructs were incorporated in to the chromosome [21]. The promoter of glyceraldehyde 3 phosphate dehydrogenase gene (P_*GAP*_) within *P. pastoris* was manipulated by a technique called random mutagenesis [22], thus exhibiting the capability of variant promoters for tight transcriptional regulation or the generation of novel regulatory transcriptional circuits. Several biotechnologically relevant therapeutic proteins have been produced by the use of synthetic promoters [23].

Major techniques for engineering heterologous production in *H. polymorpha* are based on its capability to methanol responsive growth. The promoters like formate dehydrogenase (*FMD*), and methanol oxidase (*MOX*) are the mainly exploited *H. polymorpha* promoters and is methanol inducible. Induction by methanol lead to upregulation of *MOX*, *DHAS* and *FMD* genes by 17.3, 19 and 350 fold, respectively, in comparison to glucose containing media [24]. Compared to methanol inducible promoter of *P. pastoris*, the benefit of using *H. polymorpha* is that many of them are de-repressed in glycerol containing media which is not observed in *P. pastoris* [19].

Several studies have been concentrated on metabolically and genetically engineered *H. polymorpha* species for the production of different heterologous proteins [25–27]. The biopharmaceutical production from *H. polymorpha* was enhanced after the optimization of transformation and culturing procedures along with advanced genome editing techniques. Currently used, three vaccines against Hepatitis B are synthesised by antigens obtained from bioprocess developed in *H. polymorpha*: HepavaxGene® (Johnson & Johnson), Gen Vax B® (Serum Institute of India) and Biovac-B® (Wockhardt) (http://www.dynavax.com/about-us/dynavax-gmbh/). Furthermore, pharmaceutical proteins such as insulin (Wosulin®, Wockardt), hirudin (Thrombexx®, RheinMinapharm), and IFNa-2a Reiferon® (RheinMinapharm) are effectively synthesised using *H. polymorpha* for commercial purpose [28].

Technologies for recombinant protein synthesis in *H. polymorpha* take benefit of the yeast capability to grow in methanol containing media. The promoters which are inducible in the presence of methanol like formate dehydrogenase (*FMD*), and methanol oxidase (*MOX*) are the widely used in gene manipulation techniques as it can change to methanol-substrate directed to upregulation of catabolic, for instance the *FMD* gene was about 350-fold upregulated, whereas the *MOX* and *DHAS* genes were 17.3 and 19-fold upregulated compared to glucose cultivation [24]. The integration plasmids pHIP series of *H. polymorpha* have several highly active promoters and selection markers for recombinant protein production [29].

Among the non-conventional yeast species, *Y. lipolytica* is well-researched, and identified with several functionally characterised promoters. Robust promoters such as *PICL1, PTEF1, PXPR2, PPOT1, PRPS7,* and *PPOX2* are widely used in *Y. lipolytica* [30, 31]. Nevertheless, issues like promoter repression due to low pH and elevated glucose level, or presence of promoter activity only in the existence of fatty acid by-products frequently mark these promoters unsuited for commercial purposes [32]. The *PEYK1* promoter identified from *Y. lipolytica* was found to be functional under varying erythrulose and erythritol levels of and has been anticipated to permit strict regulation and induction of protein [33]. In addition, five newly isolated promoters of *Y. lipolytica* were selected to study the consequence of gene repression due to fatty acid metabolism; and one of the selected promoters could downregulate FAD1 and OLE1 genes under depleted conditions of nitrogen. These findings provided prospective promoters that possibly will be applied for gene knock-down rather than knock-out experiments [32].

The industrial relevance of another well-established non-conventional yeast *K. lactis* relies on high expression of heterologous gene; the aforementioned could be accomplished via concentrating on the comparative potency of the native promoter. Merely less number of constitutive and inducible promoters is utilized for heterologous gene expression in *K. lactis*. The widely used inducible *LAC4* promoter system can split up the growing and gene expression stages to facilitate enhanced production or to synthesise lethal heterologous proteins. The extensively-utilized constitutive promoters of *S. cerevisiae*—*ADH1* and *PGK1* (glycolytic pathway based promoters) can be utilized for heterologous gene expression in *K. lactis* [34, 35]. The *LAC4* promoter of *K. lactis* was widely used for the synthesis of heterologous protein production and has assisted the synthesis of several industrially important proteins. This promoter is lactose inducible, but it is not fully suppressed when lactose is lacking [36]. The *LAC4* transcription is controlled by lactose or galactose induced upstream regulating regions UAS I and UAS II (Anders et al., 2006). Interleukin-1 and globulin proteins were synthesised by using the constitutive promoter—PGK in *K. lactis* [37]. Interleukin-1 is also produced by using *PHO5* promoter in *K. lactis* [38]. In another study, cellobiohyrolase promoter from *Trichoderma reesei* has been used as hybrid promoter along with LAC4 promoter of *K. lactis* for the production of GFP [39]. Engineered *LAC4* promoter with fungal signal peptide has been used for the production of human interferon β [40–42].

## CRISPR technology for gene manipulation in yeasts

CRISPR/Cas9 technique has permitted the manipulation of the Cas9 endonucleases into ‘non-active’ endonucleases (dCas9), for regulating at transcription level. Similar to the CRISPR/Cas9 system, the CRISPR/dCas9 contains sgRNA that guides the dCas9 towards the preferred sequence of the DNA. The dCas9 lacks nuclease activity and hence inhibits breakage in target DNA [43]. The dCas9 attachment on the transcription factor binding sites stops other transcription factors from binding and resulted in inhibition of transcription. CRISPRi technique established in *P. pastoris* regulates the *AOX1* gene expression, which was positioned under methanol-inducible *pAOX1* promoter. The attachment of dCas9 to the regions between − 468 and − 487 region of the *AOX1* gene resulted in its repression [44].

The CRISPR-Cas9 technology can speed up the genetic recombination in cell factories to enhance the synthesis of pharmaceuticals. CRISPR-Cas9 mediated multiplex genome editing permits to insert the resveratrol metabolic pathway into cluster with rDNA repeats of *O. polymorpha* resulting in 21 fold increase in the synthesis of resveratrol [45]. Introduction of *E. coli cadA* gene and *ALB* (human serum albumin) gene by multiple-integration allowed high product yield. Nevertheless, multiple-integration at single locus has certain challenges in creation of large metabolic pathways. Recently Schwartz et al. identified five integration locus that was appropriate for creation of large pathways in *Y. lipolytica*. The genes for lycopene biosynthesis—*crtB, crtI, crtE, HMG1* and *GGS1* were integrated into the newly identified integration sites separately enhanced the production of lycopene by 8.6 folds [46].

Among the non-conventional yeast strains, the CRISPR-Cas9 genome editing technique was first established in *K. lactis* [47]. With the help of *FBA1p* promoter *Cas9* gene was inserted at *GAL80* locus, and *ku80* gene was removed to reduce the consequence of NHEJ. The gRNA was expressed under the control of *SNR52* pol III promoter and transcription was terminated using *SUP4* terminator and an episomal structure was built by inserting another genetic element pKD1 to a 2 μ plasmid of *S. cerevisiae*. This developed gene manipulation system effectively integrated foreign DNA with flanking regions of 1 Kb on either sides to *NDT80*, *DIT1*, and *ADH1* site [47].

The thermo tolerance and rapid growth potential of *O. polymorpha*, makes it a suitable candidate for various industrial applications. A codon optimized human *Cas9* gene was introduced into a plasmid with *TEF1* promoter (*ScTEF1p*) and terminator (*ScTEF1t*) from *S. cerevisiae* to develop the CRISPR-Cas9 genome manipulation system [48]. The integrating sgRNAs—*ADE8*, *ADE12* and *PHO85* were expressed under the small non-coding RNAp *OpSNR6* promoter. This CRISPR-Cas9 system provided low gene interference efficacy. Additional introduction of a Hyg resistance expression cassette to a 60 bp homologous arms enhanced the gene disruption efficacy till 47%, that proved the reduced expression of sgRNA for directing Cas9 to targeting site. Thus, aim proved system with tRNA CUG-sgRNA fusion expression cassette was applied to enhance the sgRNA function, which considerably improved the gene disruption efficiency to 17–71% [48].

The CRISPR-Cas9 based technique was applied to form a toolkit for quick and easy strain manipulation of *S. cerevisiae* [49]. This technology for S. cerevisiae comprises 37 strong promoters, 10 protein tags, and Cas9-sgRNA constructs capable of integrating in to 23 different targeting loci. Heterologous proteins can thus be enhanced by investigating various promoters and its strength, integration loci, cultivation strategies, and protein tags for analysing recombinant protein localisation, protein stability and solubility. They also designed a web tool for CAS designing, which enables to design the oligonucleotides required for the creation of the integration cassettes. The developed technique was applied to enhance the taxadiene synthase expression, resulting in 25 fold progress in taxadiene synthesis [49].

Currently, a combination of Cas9 assisted genome editing and dCas9 mediated transcription regulation was established by manipulating *S. cerevisiae* for synthesis of naringenin, a flavonoid precursor. The Cas9 was used for insertion of a multigene controlled pathway into an intergenic site of *S. cerevisae* leading to synthesis of naringenin from phenylalanine. In another study the naringenin synthesis was enhanced through dCas9-assistedrepression of anvital gene *TSC13* to stop the synthesis of phloretic acid, a by-product [50].

## Yeast secretory pathway engineering for therapeutic proteins

The therapeutic proteins play an important role among the biopharmaceuticals used for the treatment of various diseases [51]. Proteins with therapeutic potential have had quite a great clinical impact, since they are highly specific and potent with extended effect and are less toxic [52]. After the approval of insulin produced by recombinant DNA technology [53], it became evident that therapeutic proteins have immense medical applications. Industrial scale production of therapeutic proteins is a difficult process; however microbial systems along with advanced genetic engineering techniques have extensively overcome the hurdles. Microbial systems including bacteria, yeast and algae are used for the production of proteins of human interest [54]. *E. coli* was one of the commonly exploited microbes for the production of recombinant proteins [55], however bacteria fails to carry out post-translational modifications which significantly affects the proper functioning of the desired protein. In this context, yeast has gained attention as it shows prokaryotic and eukaryotic features simultaneously and performs post-translational modification of proteins. Recombinant protein production using yeast is economically feasible due to low production cost and high titre value. Yeasts are classified under GRAS organisms and hence production of therapeutic proteins using them is quite appreciable.

*S. cerevisiae*, popularly known as baker’s yeast, is exploited widely for the production of therapeutic proteins. *S. cerevisiae* was used to produce hepatitis B surface antigen, insulin, albumin, hirudin, transferrin, glucagon and growth factors [56, 57]. Non-conventional yeasts such as *P. pastoris*, *K. lactis*, *Y. lipolytica*, *H. polymorpha* and *S. pombe* are also used for the synthesis of recombinant proteins. As the genome sequence data of these microbes are available [58], scientists can rely on the database to implement the “omics” methods such as metabolomics [59], transcriptomics [60], and proteomics [61], so as to overcome the hurdles that arise during the production of proteins using yeast. In this section, we will discuss genetic engineering tools that are used to enhance secretory protein production in yeast.

Since yeast secretes only a small number of its own proteins and its secretion pathways are similar to that of eukaryotic system, they are preferred over other organisms for the synthesis of recombinant proteins. Though the heterologous protein secretion in yeast experiences some limitations especially in glycosylation and proteolytic degradation [58], researchers had developed excellent genetic tools like glycosylation engineering, manipulation of golgi translocation, and protein folding engineering to modify the protein secretion pathway of yeast favouring its industrial application. The protein secretion is initiated by transferring the protein through the membrane of endoplasmic reticulum. For this translocation, secretion signals are required which have significant role in influencing the ultimate yield of the heterologous protein. Usually, prepro leader sequences of alpha-mating factor protein from *S. cerevisiae* are used as signal sequences of heterologous proteins. However, Kjeldsen et al., has reported that prepro leader sequences without N-linked glycosylation sites enhances secretion than that of the original sequence [62].

If the protein is misfolded prior to secretion, it will be degraded and ends in enhanced stress in endoplasmic reticulum. Hence for the proper secretion of heterologous proteins, the protein folding chaperones and redox enzymes in *S. cerevisiae* [63, 64], *K. lactis* [65, 66] and *P. pastoris* [67] are overexpressed. The folding and secretion of proteins is controlled by Hsp70 and Hsp40 families of chaperones. Zhang et al. had overexpressed Ssa1p, Kar2p, Sec63, PDI, and YDJ1p chaperones from *S. cerevisiae* in *P. pastoris*, for the better secretory expression of the recombinant protein from *P. pastoris* [68]. The expression level of anti-transferrin receptor single-chain antibody in *S. cerevisiae* was enhanced considerably by the overexpression of BiP and PDI [69]. When processing huge and complicated proteins, such as human antibodies, the folding rate in *S. cerevisiae* is inadequate. In another study, it was observed that deletion of *OPI1* causes expansion of endoplasmic reticulum resulting in reduction of stress in *S. cerevisiae*. The mutant strain overexpressing CPR5 chaperone showed enhanced antibody production [70]. Cwh41p enzyme initiates folding process by trimming N-glycan, and its overexpression in *S. cerevisiae* reduces misfolding of the heterologous protein [71]. The consecutive expression of HSR1 in *S. cerevisiae* reduces the misfolding due to the protein folding chaperones [72].

The upregulation of proteins involved in vesicle trafficking of protein from endoplasmic reticulum via golgi apparatus to plasma membrane enhances the yield of secretory proteins. It was found that overexpressing vesicle trafficking proteins Sly1p and Sec1p, accelerates the secretion of various heterologous proteins including human insulin precursor from *S. cerevisiae* [73]. GTP-binding proteins of the ARF/SAR family are important regulators of intracellular protein trafficking. These GTP-binding proteins are basically controlled by two classes of factors: guanine nucleotide exchange factors (GEFs) and GTPase activating proteins (GAPs). GAP proteins are bound to ARF proteins. Yeast homologues of ARF GAPS are Gcs1p and Glo3p. Gcs1p and Glo3p are involved in vesicle formation and their overexpression in *S. cerevisiae* expressing SEC16 improves protein secretion [74, 75].

Another hurdle is caused by the proteases expressed by yeast, as some of these are confined in the secretory pathway which ultimately cleaves the recombinant protein resulting in lower production. Protease deficient strains were developed to overcome this. The cleavage of parathyroid protein was significantly reduced when *S. cerevisiae* lacking multiple yapsin protease genes such as *YPS1*, *YPS2*, *YPS3*, *YPS6,* and *YPS7* [76]. The interruption of PRB1 and PEP4 proteases in *S. cerevisiae* enhances production of interferon β [77]. The proteolytic cleavage of fusion protein—albumin and parathyroid hormone in *P. pastoris* was reduced by deleting PEP4 and YPS1 proteases [78]. Idiris et al., has enhanced the production of human growth hormone using *S. pombe* to several folds by manipulating various genes simultaneously. Along with the deletion of seven proteases, the gene responsible for synthesizing vacuolar sorting protein—VPS10 was also deleted to increase the secretion rate of human growth hormone [79]. Human transferrin production from *S. cerevisiae* was enhanced after deleting YPS1 (reduces protein degradation) and HSP150 (eases protein purification) and overexpressing PDI1 (helps in protein folding) [80].

**Fig. 1:**
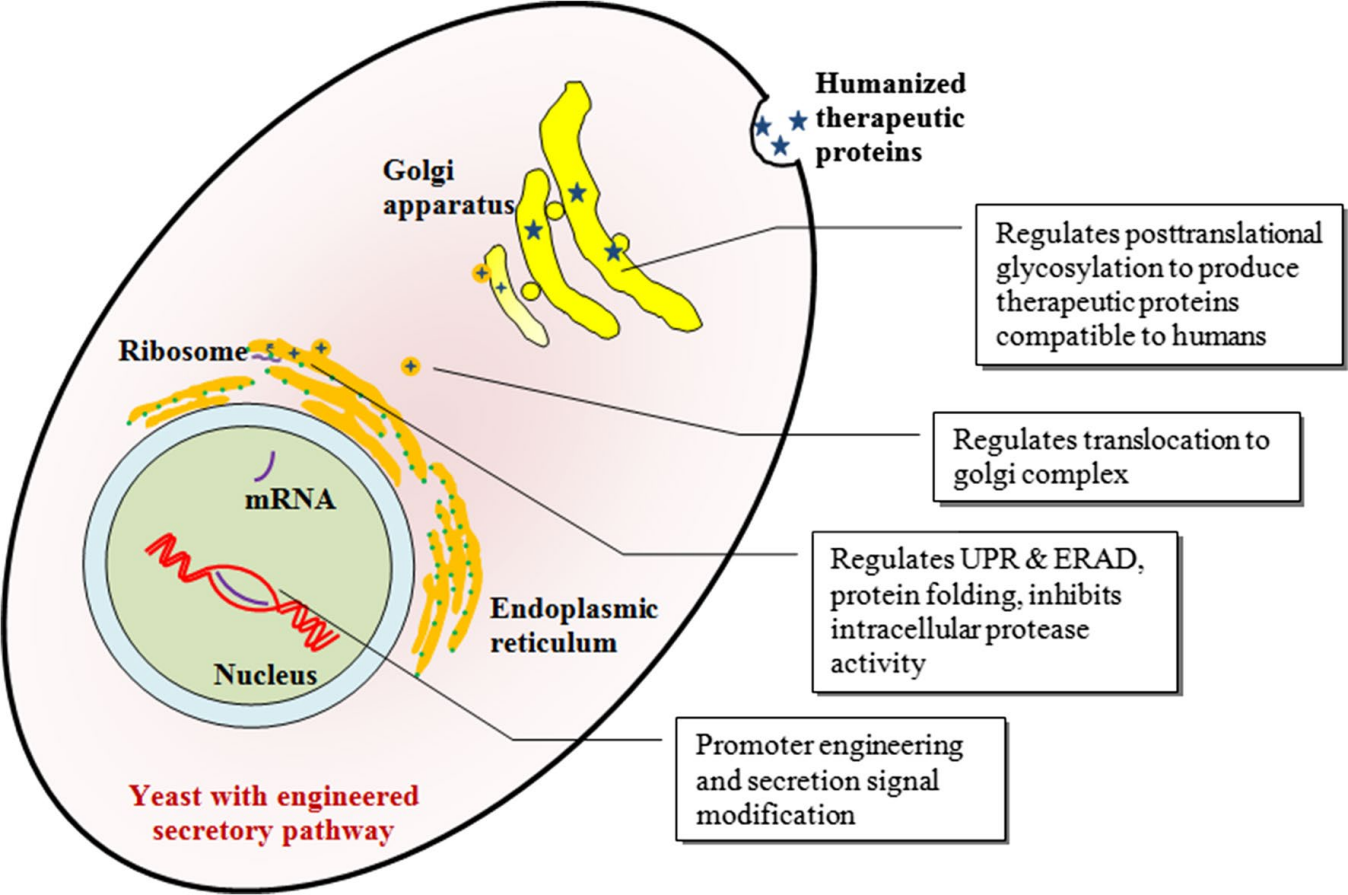
Schematic representation of engineered yeast secreting humanized therapeutic proteins

The engineering strategies adopted to modify yeast secretory pathway were reviewed in Fig. 1 and Table 2.

**Table 2:** Engineering of protein secretory pathway in yeast

Yeast strain	Gene targeted	Outcome	Reference
*S. cerevisiae*	*HSF1*	Reduce stress	[72]
	*SCJ1*	Involved in protein folding	[150]
	*PDI1*	Formation of disulfide bonds	[151]
	*SIL1*	Endoplasmic reticulum translocation	[63]
	*JEM1*	Chaperone binding	
	*HAC1*	Regulation of unfolded protein response	[152]
	*UBI4*	Post translation modification	[153]
	*CYM1*	Remove protease activity by gene disruption	[154]
	*KEX2*	Remove protease activity by gene disruption	[155]
	*VPS10*	Enhance protein secretion	[156, 157]
	*MON2*	Deletion prevents vesicle formation and enhance secretion	[158]
	*SSO*	Helps in transportation from golgi to plasma membrane	[159]
	*ALG3, ALG11*	Formation of N glycans similar to humans	[92]
	*OCH1*	Elongation during glycosylation	[160]
	*MNN4*	Mannosylphosphorylation	[85]
*S. pombe*	*PPP20*	Disruption of aminopeptidase	[79, 161]
	*ATG4*	Disruption of cysteine protease	
	*FMA2*	Disruption of methionine aminopeptidase	
	*OMA1*	Disruption of metalloendopeptidase	
	*FMA2*	Disruption of methionine aminopeptidase	
	*VPS10*	Enhance protein secretion	
	*OMH1*	Modifying O linked glycans	[162]
	*GMS1*	UDP galactose transportation	[87]
*P. pastoris*	*SSA1*	Protein folding	[67, 68]
	*SSA4*		
	*SSE1*		
	*SEC63*	Transportation of proteins	[155]
	*CUP5*		[67]
	*BFR2*		
	*KIN2*		
	*OCH1*	Decrease addition of mannose	[89]
	*ALG3*	Proper glycosylation	
	*YPS1*	Diminish protease activity	[163]
*K. lactis*	*PDI1*	Disulfide bond formation	[65, 164]
	*ERO1*	Folding of protein	[65]
	*OCH1*	Decrease addition of mannose	[88]
	*MNN1*	Decrease addition of mannose	[12]
*Y. lipolitica*	*OCH1*	Decrease addition of mannose	[88]
*H. polymorpha*	*CNE1*	Protein folding	[165]
	*ALG3*	Proper glycosylation	[86]
	*OCH1*	Decrease addition of mannose	[86]

## Glycoengineering for humanized biopharmaceuticals in yeast

Glycoengineering is a tool for enhancing and modifying the properties of proteins by altering the site for glycosylation [81]. Glycosylation is one of the important post-translational methods and accurate engineering of this result in proper folding and affect pharmacokinetic properties of the therapeutic protein [82]. Glycosylation, which is a type of post-translational modification, refers to adding various sugar molecules or polysaccharides to protein chains via specific amino acids like asparagine, serine and threonine by means of a covalent bond [83]. N-linked and O-linked glycosylation are two forms of glycosylation.

Even though yeast can do N- and O-glycosylation, there is significant difference in the outline when compared to the pattern of human glycosylation. Yeast glycosylation causes α-1,3-mannose linkages and high mannose type glycans which often results in increased immunogenicity and reduced half-life period of the synthesized therapeutic proteins [84]. Glycoengineering techniques have succeeded in developing strains that follow glycosylation pattern similar to that of humans. This is achieved in three levels of engineering to reduce hyper mannosylation, modifying mannose at N terminal and sialylation of recombinant proteins [82].

OCH1 protein commences the production of outer chain in yeasts. Man5GlcNAc2 glycan suitable for human is synthesised using an engineered *S. cerevisiae* which lacks OCH1 gene and expressing *Aspergillus saitoi* α-1,2 mannosidase gene [85]. Similarly, OCH1 deletion has been performed in *K. lactis*, *H. polymorpha*, *Y. lipolytica*, *S. pombe* and *P. pastoris* with an aim to humanize the glycosylation pathway in them [12, 86–89]. These non-conventional yeast strains produce glycans with fewer mannose and are deficient in terminal α-1,3-linked mannose residues that causes hyperimmunogenicity [58]. The deletion of OCH1 and MNN1 encoding α-1,6-mannosyltransferase and α-1,3-mannosyltransferase respectively in *K. lactis* reduces mannose addition during glycosylation and favours the production of active human granulocyte macrophage colony stimulating factor protein [12].

The glycosylation pathway in yeast is engineered to make it familiar as in human by accurately introducing human glycosyltransferases and glycosidases in yeast secretory pathway. Choi et al. has engineered *P. pastoris* to synthesize glycans similar to that of humans. This is made by deleting the gene α-1,6-mannosyltransferase and introducing α-1,2-mannosidase and human β-1,2-N-acetylglucosaminyltransferase I in *P. pastoris* [90]. ALG3 gene was deleted from *H. polymorpha* [86] and *P. pastoris* [91] to produce humanized glycoproteins. *S. cerevisiae* was engineered to synthesize N glycans with trimannosyl moiety similar to humans [92]. The potential and life time of therapeutic proteins expressed in yeast depends on the presence of sialic acids in terminal end. Hamilton et al., were successful in generating *P. pastoris* strains that produce glycoproteins carrying sialic acids in terminal position similar to humans. They have eradicated yeast glycosylation pathway by deleting 4 genes involved and introduced 14 genes to synthesize sialic acid [93].

Besides N glycosylation, O linked glycosylation is also engineered in yeast to synthesize glycans with elongated mucin having N-acetylgalactosamine bound to seriene or threonine [94] and is further elongated with the same or other sugar moieties such as fucose, galactose xylose, glucuronic acid, and sialic acid [95, 96]. *P. pastoris* was engineered to synthesize sialylated glycans by overexpressing enzymes β-1,2-N-acetylglucosaminyltransferase and mannosidase that are involved in the production of sialic acid [97]. Genes expressing core1 β1-3GalT, ppGalNAc-T1, UDP-Gal/GalNAc 4-epimerase and UDP-Gal/GalNAc were over expressed in *S. cerevisiae* to synthesize mucin type glycoproteins [98]. The human epidermal growth factor domain with terminal O-fucose was produced using genetically engineered *S. cerevisiae* expressing fusose transporter and O-FucT-1 genes from human [99].

Currently, very few biopharmaceuticals in the market are produced by yeast due to their non-optimal pattern of glycosylation. Yeasts alter glycoproteins with N-glycans enriched-mannose content in *P. pastoris* to hypermannosylated N-glycan in *S. cerevisiae*. Furthermore, *P. pastoris* could add β-1,2-mannose, and terminal α-1,3-mannoses were added by *S. cerevisiae* and both of them might be highly immunogenic [100, 101]. Recombinant proteins derived from yeast with high mannose containing N-glycans attach with respective C-type lectins receptors on the endothelial cells of liver and lymph node, macrophages and dendritic cells, which resulted in clearance from serum [102].

Even though N-glycosylation is significant in the process of protein folding of most of the recombinant therapeutic proteins; it also exercises a greater role on the pharmacodynamics and pharmacokinetics of therapeutic proteins. Therapeutic proteins with N-glycans with oligomannose-type are susceptible to fast clearance from macrophages resides in liver-Kuppfer cells by attaching to the cell surface mannose receptor [102, 103]. Pharmaceutical recombinant proteins with sialic acid terminally in glycans, display lengthier half-lives by less level of clearance [104]. Asparagine is normally the site of N-glycosylation which would be asparagine-X-serine/threonine- Asn-X-Ser/Thr situation (where X should not be proline). The primary reactions of N-glycosylation modifications are general to most of the eukaryotic organisms. Briefly, a Glc3Man9GlcNAc2 lipid-linked oligosaccharide (LLO) is assembled in the endoplasmic reticulum (ER) by several glycosyltransferases encoded by asparagine linked glycosylation (ALG) genes. This predecessor is transferred co-translationally to a new chain of amino acid by a complex of oligosaccharyltransferase. Glucosidase I and II perform N-glycan deglucosylation and resulted in the synthesis of a monoglucosylated structure which can bind calnexin or calreticulin and helps in protein folding. Consequently, α-1,2-mannosidase residing in ER eliminates α-1,2-mannose. Additionally, Htm1p in the ER process α-1,2-mannosidase and could uncover the terminal α-1,6-mannose residue, which act as a signal for protein degradation and proteins do not attain appropriate folding. Appropriately folded proteins are conveyed to the Golgi bodies, and their N-glycans are additionally processed in a precise way.

Yeast glycoengineering includes the elimination of glycosylation specific to yeast, occasionally pursued by the synthesis of hybrid human type glycans. Subsequent to the alteration of the of N-glycan type on the recombinant protein, significant struggle has been dedicated to decreasing the macro and micro heterogeneity in glycoproteins. To reduce macro heterogeneity, the basic variety of recombinant proteins created by unusual activity of N-glycosylation sites, various approaches have been attempted to enhance the efficacy of co-translational transfer of N-glycan to glycoproteins. Another technique includes controlling the dolichol pathway flux.

The very first step of humanizing yeast for N-glycosylation is the elimination of the mannose rich residues and hypermannosylated compositions, either by the removal of glycosyltransferases or by blocking the assembly of the lipid linked oligosaccharide. Another strategy is the incorporation of various glycosyltransferases and glycosidases to create hybrid N-glycans. Another approach is the introduction of endo-β-N-acetylglucosaminidase which proficiently converts yeast N-glycosylation which removes high-mannose N-glycans and produces deglycosylated product. The incorporation of N- and O-glycosylation pathways similar to humans in yeasts resulted in the expression of glycoproteins improved with complex N-glycans or humanised O-glycans [105].

In yeast glycoengineering, N-glycan humanization mainly takes place in three steps. Firstly, yeast hypermannosylation is to be limited or eliminated through pathway manipulation perturbations. One of the method is to interrupt or delete glycotransferase OCH1 genes and expressing mannosidase enzyme, resulting in Man5GlcNAc2 (human-like) glycoform [106]. In another strategy, ALG3 gene was removed and various modifications were done to attain humanised Man3GlcNAc2 glycoform [106]. In the subsequent stage, further alteration of terminal mannose residue is attained using N-acetylglucosaminetransferase, GnT-I, and formed glycoform with terminal GlcNAc. The addition of another GlcNAc moiety to mannose is executed using GnT-II gene, resulted in GlcNAc2–Man3GlcNAc2. The final step is carrying out sialylation of those humanised glycoforms by the incorporation of heterologous genes to synthesise sialylated glycoproteins with 90% or more terminal sialylation [93].

## Fermentation and scale-up approaches for pharmaceutical protein production in yeast

Recent advances in omics technologies and metabolic engineering have made incredible improvements in the area of biopharmaceuticals synthesis by recombinant yeast. But the commercial scale synthesis of pharmaceutical proteins is still at initial stages. The major step after recombinant strain construction and pathway engineering is the optimisation of strain cultivation conditions for the process scale up, strain improvement through screening, growth kinetics, and production kinetics, optimizing biomass growth and developing suitable fed batch production processes. Various attempts have been tried to increase the protein synthesis by optimising media, temperature, pH, inoculum and production kinetics [1]. Carbon substrates were optimized in various studies to reduce the production cost and also for improved biosynthesis. Finding an appropriate growth media was also vital to enhance the production. This section focuses on specific features of heterologous protein synthesis related to deviations in biomass growth and their consequences for strain construction and screening, as well as on the concept of rational evaluations between culturing systems for the designing of particular bioproduction techniques in bioreactors.

The methylotrophic yeast *P. pastoris* is well known for its very high growth rate, high cell concentrations during fermentation, its capability to produce huge amounts of properly refined prokaryotic or eukaryotic recombinant proteins (intracellular or secreted) consisting of *AOX1* promoter [107]. *P. pastoris*, as a protein production system has an enormous future significance as it is already known to produce a wide range of proteins [108]. Here, we have considered the methylotrophic yeast *P. pastoris* to discuss the scale up strategies for pharmaceutical protein production as *P. pastoris* is very well known for its very high growth rate, high cell concentrations during fermentation and its capability to produce huge amounts of properly refined prokaryotic or eukaryotic and humanised recombinant proteins.

### Growth kinetics of recombinant strains during heterologous protein production

*P. pastoris* are capable of growing in glucose, glycerol and methanol as they are the preferred carbon sources in industrial processes. During batch mode of fermentation, where surplus substrate is present, direct assessment of unhindered biomass growth features is possible. *P. pastoris* exhibits better growth when methanol is used as carbon source instead of glucose or glycerol. Basically diauxic performance is present in batch cultures. That means glucose or glycerol supress the use of methanol and these carbon sources are then used successively. Strains expressing recombinant proteins often display maximum specific growth rates considerably less than those detected with a non-recombinant host strain. Maximum specific growth rate is one crucial factor pointing out the altered and stressed cell physiology during recombinant protein production. Specific growth rate (μ_max_) determination is hence an inevitable step before commencing strategies for method improvement. Engineered Mut+ strains of *P. pastoris* are found to grow in methanol containing media at growth rates from 0.028 to 0.154/h, and strains with MutS in a narrow collection from 0.011 to 0.035/h. Hence, fedbatch with well-controlled environments, the same lower μ-value can be used to regulate any of MutS or Mut+ organisms. The subsequent benefit in production processes involving MutS organisms is their vigour in tolerating and managing methanol overfeeding [109–111].

There is a rising attention in the pharma industry to improve biosynthetic efficacy via shifting batch to continuous biomanufacturing. Rahimi et al. established that under similar process condition, continuous fermentation of *P. pastoris* Mut+ expressing hepatitis B surface antigen (HBsAg) has remarkably greater proficiency compared to the fed-batch process. In another work same group evaluated various dilution rates, process efficiency, and also assessed several strain-specific parameters. According to them, continuous process at the dilution rate of 0.015 L/h confirmed the maximum efficacy compared to other dilution rates. In the optimised dilution rate, the HBs antigen titer was 4.26 mg HBsAg/L/h, respectively [112].

### Process kinetics of recombinant protein production

The biomass specific growth rate of (μ) is a key factor in enhancing product synthesis [113]. The association between specific productivity and growth rate affects the balance between several mechanisms such as protein folding and trafficking in a cell until the protein is secreted. The association is termed as production kinetics and is significant in strategic designing to maintain ideal growth rate by adding the precise carbon-source in fedbatch manner [114]. *GAP*-promoter controlled synthesis of secreted proteins are found to be growth associated as it typically proportionately increases with rise in specific growth rates till it reaches near to the respective specific growth rate (μ_max_)values. Similar to *GAP*-controlled kinetics, it would appear ideal to frame better approaches for process development at growth rates close to maximum specific growth rate (μ_max_). Nevertheless, slow controlling of μ in a reducing manner over time during cultivation *P. pastoris* for recombinant protein production is recommended as this can result in best titres and best productivities possible. Unlike *GAP*-controlled kinetics, the kinetics of *AOX1*-controlled protein production is bell-shaped. The bell shape may be correlated to saturation of the secretory pathway as a result of high expression levels.The bell shape may also be due to using methanol as substrate, which is less advantageous [115, 116]. Inspite of the current information gap for *AOX1*-controlled metabolite synthesis, design of processes on a lesser specific growth rates lesser than 0.04/h is found to be appropriate for achieving high titres and productivities [115–117].

### Screening of recombinant strains

The highly efficient recombinant clones are usually recognized at primary phase of process development, and is then cultivated all through the remaining developmental stages of the manufacturing of a recombinant protein. Normally, high-throughput screening techniques are adopted to manage for noteworthy clonal variations (which is mainly observed in recombinant strains) and to decrease the substantial performance load in bioreactor experiments. Thus screening of recombinant clones is a key step in the production of heterologous proteins. Advent of high throughput technologies revolutionised the field of recombinant clone screening technologies allowing parallel screening of several batch experiments using 96-deep-well plates or similar technologies [111, 118].

In case of pulsed screening of recombinant *P. pastoris* in deep welled plates, the obtained optimum growth rate values for product formation using methanol (*AOX1* controlled) as substrate were lesser than the maximum specific growth rates obtained, but in GAP controlled product formation is normally near to μ_max_ [115, 116]. Hence, optimal efficacy of Mut+/*AOX1*-strains could not be attained in batch cultures, and clusters of clones with various levels of productivity should be selected for subsequent experiments [111].

The screening data obtained from *GAP*-promoter controlled strains in batch cultures can be scaled up and transferred to fed batch, as the optimum production values are close to μ_max_ values [117].

### Biomass growth characteristics and product formation

The compulsory first stage for any product development process is characterisation of strains physiologically. The maximum specific growth (μ_max_) rate specific to a strain is a key criteria limiting maximum conceivable feed added in order to prevent accumulation of substrate. For determining μ_max_, recurrent pulses of 1% methanol in batch cultures were reported to be simple and suitable method to follow. Product synthesis kinetics knowledge, and the specific productivity q_p_(μ)-relationship, are vital to the construction of a fedbatch synthesis technique in which strain cultivation is regulated at a particular μ-value which is less than μ max by the incorporation of carbon-source in a controlled manner [117]. Native strain of *P. pastoris* is capable to grow on several carbon substrates and energy sources [119], of which glycerol, glucose and methanol are the most widely used in industrial processes. The selection of carbon source and, therefore, the feasible working range with respect to specific growth rate (μ) and optimum productivity (q_p_) is reliant on the selected promoter. In the scenario of *AOX1* promoter-regulated synthesis of product, fedbatch growth at pre-set specific growth rates of 10, 30 and 50 percentage of μ_max_, can be attained by exponential incorporation of carbon substrate, which is to be held stable throughout the entire time of cultivation [120]. A similar systematic process development involving three exponential fed batch techniques would permit an early and effective calculation of kinetics of product formation, also circumventing need for further production and variations in the ultimate outcomes. Simple cyclic fed-batch culture (cfbc) has been applied to *P.pastoris* for human serum albumin production. The developed process comprising continuous feeding of media with intermittent drawings of culture resulted in a protein yield of 13.4 mg per gram of biomass. In cfbc, product yield was eventually restricted by the rate at which the strains might assimilate methanol [121]. In another study an insulin precursor coding gene was codon-optimized for heterologous protein expression in *P. pastoris*, and introduced into the genome *P. pastoris* strain under the control of *AOX1* promoter. The strain could grow large-cell density in a batch process using a definite medium with low salt and elevated concentrations glycerol. After the batch growth, synthesis of insulin precursor was performed at a methanolconcentration of 2 g/L and kept as constant throughout the cultivation process. This vigorous feeding technique led to the synthesis of ~ 3 g IP per liter of culture fluid [122]. Fedbatch experiments at 25, 50 and 75 percentage of μ_max_ are suggested for fully growth-associated, *GAP*-controlled recombinant protein production [117, 120] Applications of dynamic fed batch feeding strategies can be considered to be very encouraging and time saving in the long run. However, more expertise and experience in generating consistent, repeatable and reproducible results are required [111].

### Fed-batch process for the cultivation of recombinant proteins

Factors such as specific productivity, biomass in the reactor, and time required for production are crucial in influencing the performance of bioprocess manufacturing technologies whose target is to produce high quality and high quantity product within short time durations. Normally, a large initial biomass concentration and a low specific growth rate during manufacturing phases are advantageous. Appropriate methodologies for highest titre and utmost metabolite formation show a trend in which there is high specific growth rate initially for rapid growth of biomass to higher concentrations. Then, there is a successive decrease in specific growth rate in production stage and maximum productivity is achieved at optimum growth rate condition [117].

*Y. lipolytica* combines together the characteristics of prokaryotes (in terms of genetic engineering and simplicity of growth) and eukaryotes (in terms of post-translational modifications, protein folding and assemblies) to stand out as a unique host system [123]. For *Y. lipolytica,* most studies give optimized results by using the fedbatch method. The fedbatch strategy of cultivation combines the possibility of separating or differentiating growth and production stages ensuring good cellular activity by means of controlled nutrient utilisation. Fedbatch strategy prevents over-nutrition and starvation of cells. A higher or lower concentration of nutrients is also not desirable as it could transform the physico chemical properties of cell culture media, negatively impact cell growth and protein manufacturing mechanisms.

Towards the end of batch stage when the major carbon source has been used up, nearly all fedbatch methods used to begin with feeding either the complete culture medium [124, 125] or only the respective carbon source [126, 127]. This strategy ensures cell growth maintenance, uninterrupted metabolic activity cycles including protein generation pathways. In processes where cell growth and protein generation stages needs to be separated, such a type of fed-batch feeding can be performed only during growth phase [128]. Nonetheless, opting for nutrient feeding throughout production phase can deliver higher protein biomass and the transition of cells growth-to-production phase becomes smooth [129]. New modelling methods to supervise process productivity of *P. pastoris* was developed recently [130] this might be used in future for *Y. lipolytica* as the genome level metabolic models of this yeast can be obtained [131, 132].

Inducible promoters are utilised in fedbatch methods to separate the growth and production phases, so as to reduce the metabolic freight which the protein biosynthesis passes on to the host cells [133]. Initially, the cultivated biomass is endorsed to grow to achieve a concentration that can sustain the successive protein manufacturing cycles [129, 134]. Feeding of an inducer to the culture medium slows down the cell growth. It can be advantageous for protein synthesis as the metabolic fluxes are activated. This method was comprehensively executed for proteins of human origin to poise the damaging impact on cell health triggered by instigation of human gene expression [129, 135].

## Bioprocess development in non-conventional yeast for enhancing recombinant proteins

Along with cell engineering strategies, several cultivation techniques are being developed to enhance the synthesis of heterologous biopharmaceuticals. Human lysozyme synthesis in *K. lactis* K7 was enhanced considerably by adjusting the media constituents and presenting a biofilm reactor. Lactose (16.3%), yeast extract (0.8%) and casamino acid (1.2%) was considered as the optimal growth media composition for large scale lysozyme synthesis. The biofilm reactor offers an inert immobilisation of *K. lactis* on solid support and might resulted in enhanced product synthesis [136]. The synthesis of heterologous staphylokinase in *O. polymorpha* was enhanced by introducing an effective fermentation technique. A concise period fermentation technique of 80 L volume was designed for *O. polymorpha*. The key process factors such as feeding technique, temperature, media constituents and pH were adjusted. The maximum product yield attained was 1 g rSAK/L [137].

In *Y. lipolytica*, cultivation medium and mainly C/N ratio have been confirmed to be key features for the synthesis of numerous recombinant proteins [138, 139]. Nitrogen starvation can stop the TCA and activate lipogenesis via ATP citrate lyase, considerably changing the supply of flux in the strains [140]. Thus, Saez-Saez et al. [141] recently enhanced the recombinant resveratrol titers of *Y. lipolytica* ST9671 by evaluating several growth media (mineral medium, YNB, and the rich medium YP) with varying C/N ratios. Growth in mineral medium caused 33–181% high product titers compared to YNB medium, when evaluating the trials with the same carbon resources. In controlled fed-batch fermentor, maximum titer was obtained as the strain synthesised 12.4 g/L recombinant resveratrol. Furthermore, the bioprocess was conducted in a low-cost mineral medium devoid of any high-priced aromatic intermediate supplements.

## Conclusion

The model organism yeast exhibits the properties of both prokaryotic and eukaryotic organisms which makes them preferred over other microbial systems for the production of compounds, especially proteins with therapeutic applications. However, there are some limitations in yeast system which many a time reduces the quantity and quality of desired protein of our interest. With the advancements in the molecular biology techniques, the researchers were able to modify the yeast system and make them efficient to produce more or less humanized products. Along with *S. cerevisiae*, the non-conventional yeast systems such as *K. lactis*, *P. pastoris* were engineered to enhance the production of heterologous proteins. These modified systems are gaining more attention considering their potential to synthesize products compatible for human. The different steps of recombinant protein production in yeast, from start to end, can be engineered in a successful manner by improving fermentation methods, expression systems, and introducing synthetic biology techniques. Through the years the ability to identify strains and introduce desired genes of our interest under active promoter system with proper secretion signals was achieved. Now it is possible to control intracellular proteases and ubiquitination which degrade the recombinant proteins, reduce the stress an increase space in endoplasmic reticulum, proper folding and help in translocation to golgi complex. The improvements in glycosylation engineering make it possible to produce humanized compounds that have better half-life and less immunogenicity. However, more innovative engineering techniques should be implemented in the synthetic and secretory pathways of the yeast system to produce therapeutic proteins.

## Data Availability

Not applicable.
